# Implementation of a pan-European ecosystem and an interoperable platform for Smart and Healthy Ageing in Europe: An Innovation Action research protocol

**DOI:** 10.12688/openreseurope.14827.1

**Published:** 2022-07-04

**Authors:** Katja Seidel, Melanie Labor, Richard Lombard-Vance, Emma McEvoy, Michael Cooke, Lucia D’Arino, Deirdre Desmond, Delia Ferri, Philip Franke, Ilenia Gheno, Sonja Grigoleit, Barbara Guerra, Artur Krukowski, Marketa Pešoutová, Ilia Pietri, David Prendergast, Rebecca Maguire, Marco Manso, A. Jamie Saris, Sari Sarlio-Siintola, Tatiana Silva, Eleni Zarogianni, Malcom MacLachlan, SHAPES Consortium

**Affiliations:** 1 Department of Anthropology, ALL Institute, National University of Ireland, Maynooth, Maynooth, Co. Kildare, Ireland; 2 Department of Psychology, ALL Institute, National University of Ireland, Maynooth, Maynooth, Co. Kildare, Ireland; 3 School of Law and Criminology, ALL Institute, National University of Ireland, Maynooth, Maynooth, Co. Kildare, Ireland; 4 Edward M Kennedy Institute, Department of Psychology, ALL Institute, Social Sciences Institute (MUSSI), National University of Ireland, Maynooth, Maynooth, Co. Kildare, Ireland; 5 World Federation of the Deafblind, Las Palmas, Gran Canaria, Spain; 6 School of Law and Criminology, Centre for European and Eurasian Studies, ALL Institute, National University of Ireland, Maynooth, Maynooth, Co. Kildare, Ireland; 7 Carus Consilium Sachsen GmbH, Dresden, Saxony, 01309, Germany; 8 AGE Platform Europe, Brussels, 1150, Belgium; 9 Fraunhofer Institute for Technological Trend Analysis INT, Fraunhofer Gesellschaft zur Förderung der angewandten Forschung E.V., Munich, Bavaria, Germany; 10 EDGE, Edgeneering LDA, Lisbon, 2500 218, Portugal; 11 ICOM, Intracom SA Telecom Solutions, PEANIA, 19002, Greece; 12 Palacký University Olomouc, Olomoucký University Social Health Institute, Olomouc, 771 11, Czech Republic; 13 Department of Psychology, ALL Institute, Human Health Institute, National University of Ireland, Maynooth, Maynooth, Co. Kildare, Ireland; 14 Department of Research and Development, Laurea University of Applied Sciences, Vantaa, 01300, Finland; 15 Department of Research and Development, Treelogic - Tree Technology SA, Madrid, 28020, Spain

**Keywords:** Healthy ageing, older adults, integrated care, digital technologies, assistive technologies, medical devices, open ecosystems, standardisation, interoperability, scalability, socio-technical systems, care, quality of life, ethics, data protection, ethnography, transdisciplinary

## Abstract

As life expectancy continues to increase in most EU Member States, smart technologies can help enable older people to continue living at home, despite the challenges accompanying the ageing process. The Innovation Action (IA) SHAPES ‘Smart and Healthy Ageing through People Engaging in Supportive Systems’ funded by the EU under the Horizon 2020 Research and Innovation Programme (grant agreement number 857159) attends to these topics to support active and healthy ageing and the wellbeing of older adults.

This protocol article outlines the SHAPES project’s objectives and aims, methods, structure, and expected outcomes. SHAPES seeks to build, pilot, and deploy a large-scale, EU-standardised interoperable, and scalable open platform. The platform will facilitate the integration of a broad range of technological, organisational, clinical, educational, and social solutions. SHAPES emphasises that the home is much more than a house-space; it entails a sense of belonging, a place and a purpose in the community. SHAPES creates an ecosystem – a network of relevant users and stakeholders – who will work together to scale-up smart solutions. Furthermore, SHAPES will create a marketplace seeking to connect demand and supply across the home, health and care services. Finally, SHAPES will produce a set of recommendations to support key stakeholders seeking to integrate smart technologies in their care systems to mediate care delivery.

Throughout, SHAPES adopts a multidisciplinary research approach to establish an empirical basis to guide the development of the platform. This includes long-term ethnographic research and a large-scale pan-European campaign to pilot the platform and its digital solutions within the context of seven distinct pilot themes. The project will thereby address the challenges of ageing societies in Europe and facilitate the integration of community-based health and social care. SHAPES will thus be a key driver for the transformation of healthcare and social care services across Europe.

## Plain english summary

European citizens are living longer, with life expectancy exceeding 80 years in most European states. This increase in life expectancy is creating new challenges for health and care systems as older adults are at greater risk of cognitive impairment, frailty, and multiple other chronic health conditions. Such impairments can have negative consequences for older adults’ quality of life and independence, also placing burdens on those who care for them. Digital care technologies have the potential to improve integrated care for older adults, allowing them to live independently with multiple chronic health conditions, by monitoring their health and wellbeing, assisting with care, and creating smart home environments. SHAPES aims to empower older adults by developing and testing a range of digital technologies to facilitate long-term healthy and active ageing and the maintenance of a high-quality standard of life. 

## Introduction

Life expectancy across Europe has increased. According to Eurostat (2021b), in 2018 the EU-28 the average life expectancy for a 65-year-old person was twenty years. However, longevity does not guarantee health, nor the absence of impairment or disability ( OECD & EU, 2020). In the EU, people aged 65+ may live more than half of their remaining years with increasing frailty or with a disability ( European Commission, 2013). Particularly in the last ten years of life ( Eurostat, 2021a), Europeans often experience functional and sensory health limitations ( Eurostat, 2021d) and chronic diseases ( Eurostat, 2021c), reducing quality of life and independence. This also presents a challenge to the sustainability of health and care systems (e.g., Thomson *et al.*, 2009).

The diversity of Health and Care (H&C) systems in European countries and regions adds to the challenge. Health literacy, technology and individual involvement in care are key to making H&C more user-friendly and empowering. The redesign of care delivery suggests a benefit in shifting care from hospitals to home and the community ( Prendergast, 2020) and citizens, including older individuals, must be actively involved in all levels of health care decision-making ( International Alliance of Patients’ Organisations, 2014).

Responding to the DT-TDS-01-2019 “Smart and healthy living at home”, the Horizon 2020 *Smart and Healthy Ageing through People Engaging in Supportive Systems* (SHAPES) project will seek to support older adults in retaining their independence and autonomy alongside the occurrence of any age-related limitations they may face. SHAPES is a four-year project (November 2019 – October 2023), which will create a unique and open pan-European Ecosystem including a standardised, interoperable, and scalable Platform for the integration of smart technologies. The SHAPES Platform has the following three core functions: to collect and analyse data relating to individuals’ health, environments, and lifestyles; to identify needs on an ongoing basis; and to provide reliable, trustworthy, and affordable recommendations and solutions in return. SHAPES Digital Solutions specifically address users' requirements and expectations on the use of innovative technologies to support and extend older adults’ independent living and active and healthy ageing at home.

**Figure 1.**
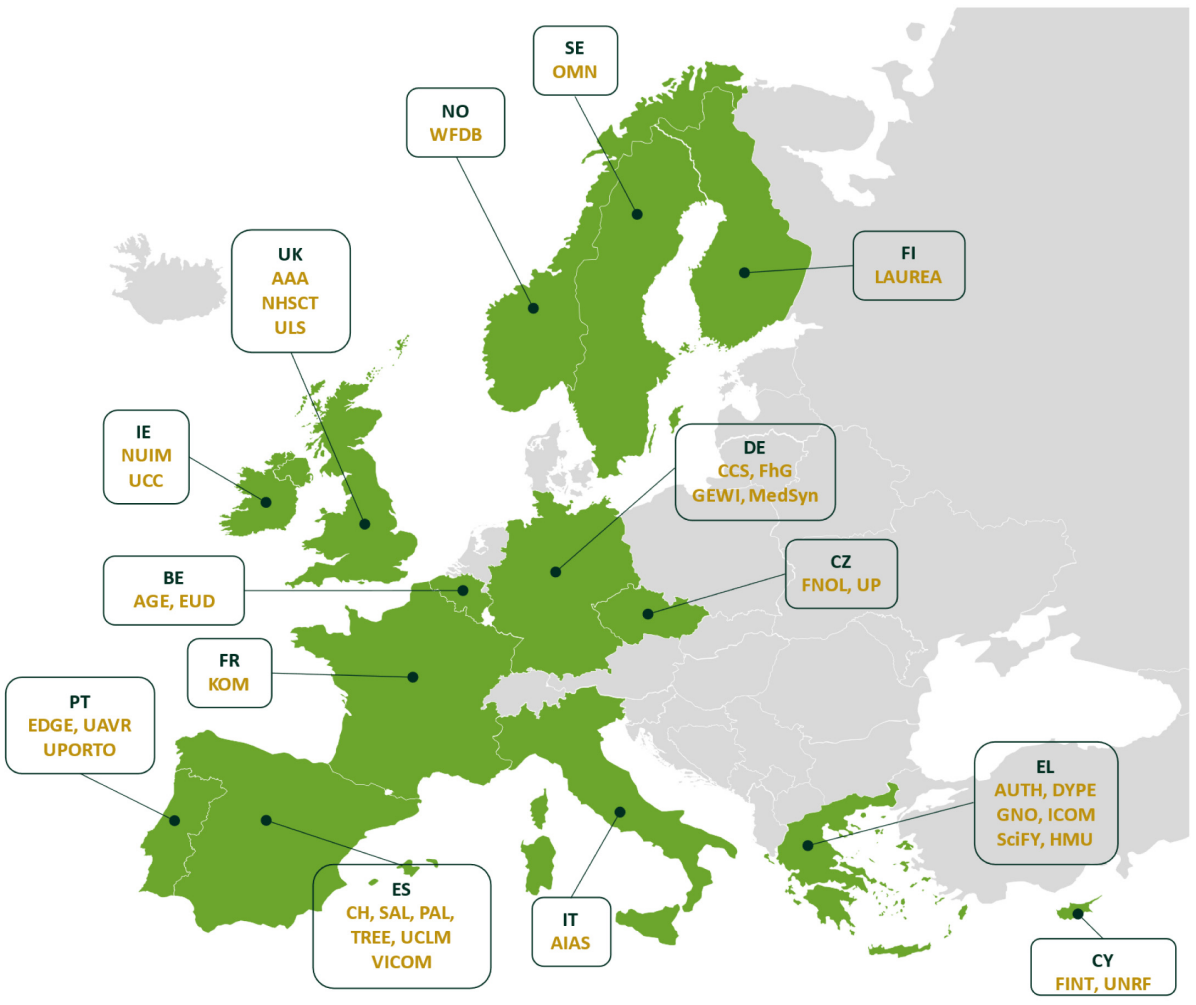
SHAPES Consortium countries.

**Figure 2.**
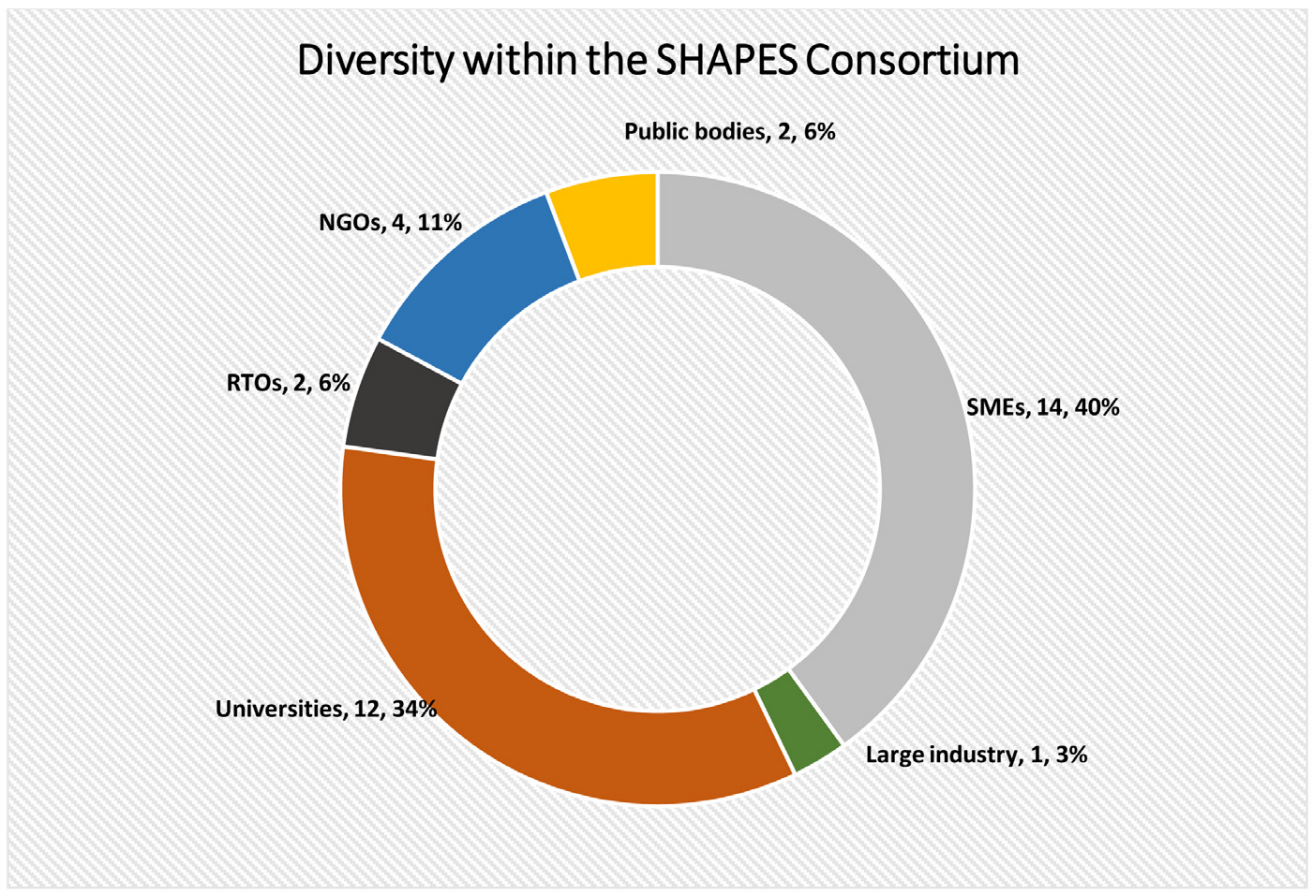
Breakdown of fields of expertise within the SHAPES Consortium.

The SHAPES Consortium ( Figure 1) consists of 36 partners from 14 countries (Ireland, Belgium, France, Germany, Greece, Italy, Norway, Czechia, United Kingdom, Finland, Spain, Portugal, Cyprus, Sweden) from a diverse range of backgrounds (see Figure 2) including academia, research and technology organisations (RTOs), industry, public bodies, and non-governmental organisations (NGOs). More specifically, the Consortium consists of twelve universities (MU, AUTH, FNOL, LAUREA, UP, HMU, UCLM, UAVR, UPORTO, UCC, UNRF and ULS), fourteen small-medium enterprises (SMEs) (AAA, AELTD, CCS, CH, EDGE, FINT, GNO, KOM, MedSyn, OMN, PAL, SAL, SciFY, TREE), one large industry partner (ICOM), three RTOs (FhG, GEWI and VICOM), two public bodies (NHSCT, DYPE5), and four NGOs (AGE, AIAS, EUD and WFDB) (see Appendix I for more details on these organisations).

The SHAPES Platform will be co-developed based on the following empirical research areas: the lifeworlds of older adults; the organisational, structural, and sociotechnical factors associated with the delivery of integrated care services; a set of technological requirements to accommodate an open and interoperable ecosystem of digital solutions specifically tailored to support and extend independent living of older individuals. The SHAPES Platform will then be validated through a multidisciplinary, large-scale piloting campaign encompassing the following seven key themes:

1) *Smart Living Environment for Healthy Ageing at Home*,
2) *Improving In-Home and Community-based Care*,
3) *Medicine Control and Optimisation*,
4) *Psycho-social and Cognitive Stimulation Promoting Wellbeing*,
5) *Caring for Older Individuals with Neurodegenerative Diseases*,
6) *Physical Rehabilitation at Home, and*
7) *Cross-border Health Data Exchange Supporting Mobility and Accessibility for Older Individuals*.

These pilots will involve over 2000 older adults across fifteen pilot sites in 10 states (nine EU-28 Member States (MS) and Northern Ireland), including six reference sites of the European Innovation Partnership on Active and Healthy Ageing (EIP on AHA). In addition, SHAPES will draw on expertise of professionals from a wide range of disciplinary and professional backgrounds within and outside of the consortium. Crucially, SHAPES will build and operate within clearly defined ethical and legal frameworks to protect both researchers and participants’ fundamental rights, privacy and personal data, while retaining the latter’s trust. Alongside user requirements, ethical requirements are particularly important when developing and employing solutions linked to fundamental rights and when the target group is older adults.

The insights gathered throughout the lifetime of SHAPES will make a valuable contribution towards improving the lives of older adults in Europe and towards enhancing the sustainability of health and care systems in Europe. The Action will contribute to the work of the World Health Organisation’s Digital and Assistive Technology for Ageing (DATA) initiative ( Khasnabis *et al.*, 2020). The research activities in SHAPES will result in a clearer understanding of the lived experiences of older adults, and of the structures and processes governing European health and care systems. Furthermore, the piloting activities will provide EU policymakers and industry with validated, cost-effective, trustworthy, and innovative solutions to support active and healthy ageing. It will increase our understanding for the implementation of socio-technological, smart innovations for healthy ageing and the acceptance of and needs for digital health applications by older adults. Based on the validation activities of the suite of digital solutions, SHAPES will formulate governance and business models for practical applications beyond the project, which will be responsive and adaptable to societal and technological changes.

## Goals and value proposition of SHAPES

SHAPES aims to facilitate long-term healthy and active ageing and the maintenance of high quality-of-life for older adults in Europe. As people live longer, the frequency and risk of injuries, frailty and long-term chronic illnesses increases, as does the risk for isolation and loneliness ( Drennan *et al.,* 2008). At the same time, societies will benefit from older adults’ contributions to addressing societal needs and informal sectors if they remain healthy and agile and have access to resources and social relations ( Buch, 2015). The combined expertise of the multi-disciplinary SHAPES consortium will enable us to study and understand the lifeworlds of older people and to develop and integrate meaningful user-centred digital solutions and services.

### Overview of SHAPES aims

Responding to the challenges associated with longer lifespans, SHAPES seeks to foster good health, improved care systems, and participation in society through its open and interoperable platform that creates integrated networks of people, data, and resources. It envisions providing and integrating smart and easy-to-use services that help older people to continue living independent, active, and healthy lives – and help their formal and informal carers to assist them in living as such. SHAPES thereby is aligned with efforts of the EU to mitigate the (negative) effects that the ageing population will have on health and care systems. This will involve new models of long-term support and care for older individuals, strong cooperation among all stakeholders of health and care, services that assist professional and informal caregivers, and integrated and economically sustainable tools.

### SHAPES and inclusive access

The SHAPES platform seeks to provide inclusive access to a broad range of technological, organisational, clinical, educational, and social solutions that have the potential to foster wellbeing and quality of life. It will mediate digital services and care models that put greater emphasis on inclusivity and accessibility. Furthermore, SHAPES will produce recommendations to promote the interaction between in-home and local community environments, and health and care networks. Concentrating on the needs of care receivers, integrated care involves the coordination of the processes between diagnosis and treatment, between primary care and secondary care, and between different therapeutic fields of expertise (see e.g. Flanagan *et al.*, 2017; Frost *et al.*, 2020; Looman *et al.*, 2019; Platzer *et al.*, 2020; Rocks *et al.*, 2020). Putting the user at the centre in all these areas, the digital technologies piloted in SHAPES need to fulfil a range of requirements, including user-, business-, security and technical requirements and finally, ethical and legal requirements. In so doing, the SHAPES approach will provide high-quality integrated and seamless care directly in the homes of older users.

The shift from hospital-based to community-based care is a global trend. For example: Denmark is leading the world in shifting care from hospitals to home ( Rostgaard, 2006), and Sweden and France have also seen significant increases in in-home care as a result of recent public policies ( Jönsson *et al.*, 2011). Individuals with chronic conditions need regular care and/or support that can often be delivered at home by care professionals (e.g., community nurses) who coordinate with the individual’s clinicians, potentially using information and communication technologies (eHealth) such as remote monitoring devices that can help minimise the risk of (re)admission to care homes and hospitals and thereby enable substantial cost savings.

### SHAPES and service digitisation

The evolution of digitisation of services and eHealth technologies are key to restructuring care delivery and supporting personalised and customised care. Developing better communication tools between clinicians and individuals, using e-solutions for managing patient records and giving individuals due ownership of their records, as well as e-Health initiatives such as ePrescription, contribute to improving coordination between individuals and H&C providers so that care can be brought into the home. Central to independent living is the recognition that specifically designed, ICT-based assistive technologies can be of great benefit to older individuals who are increasingly at risk of impairment pertaining to physical, mobility, sensory, and cognitive performance. Developments in ICT-based home care, including ways of monitoring wellbeing and providing a secure home environment, and key emerging technologies on robotics and smart sensors open up the concept of Ambient Intelligence and offer the potential for different environments (at home, in local neighbourhoods, during transportation) to embed intelligence that helps with everyday practices. However, European measures remain modest, with experiments involving advanced ICT services – from smart home technologies to eHealth solutions – supporting H&C through small-scale, localised initiatives. SHAPES takes up these propositions and provides a pan-European Platform, comprising digital and technological solutions, a governance model framework, and real-life insights from relevant stakeholders. In relation to these key objectives, the project aims to construct an interoperable Platform integrating smart digital technologies which will collect and analyse data regarding older people’s health, environments, and lifestyle. This information will help to identify their needs, and to provide personalised solutions. SHAPES, which relies on the collection and analysis of personal and sensitive data, such as a person’s health information, takes a rigorous approach to ensuring that personal data are protected at all times.

### SHAPES digital technologies

SHAPES digital technologies aim to support older people as they embrace independent living and active and healthy ageing at home. They will cover a wide spectrum of assistive technologies such as assisted living platforms, online communication and accessibility tools, cognitive stimulation and rehabilitation programmes, conversational assistants and chatbots, robots, telehealth and remote monitoring platforms, security solutions and COVID-19 response tools, as well as data analysis solutions in the domains of predictive systems, anomaly detection and wellbeing assessment. All of these will be brought together in a European open ecosystem that enables the large-scale deployment of a broad range of digital technologies. In order to achieve these ambitious objectives, the qualitative research and piloting activities of the project engage with over 2,000 older individuals and involve hundreds of key stakeholders.

### SHAPES lifeworlds and digital inclusion

SHAPES recognises that a technology platform for smart and healthy ageing has to work in different ways for different people and their lifeworlds. *Lifeworld* is conceptually understood as the immediate experiences, activities, and contacts that make up the experiential world of an individual’s life. It is the “given”, common-sense world of subjects in and beyond the physical environments, including a manifestation of collective meanings and ways of being in the world (see also Hallowell, 1960; Jackson, 2017; Schütz, 1945). The empirical ethnographic assessment of the lifeworlds of older adults across Europe will thus build awareness of the importance of cultural, social, ethnic, and gender diversity of older adults, as well as the range of disabilities and dependencies that may be experienced by older adults. Based on the empirical data gathered in ten pilot sites and replicating sites, SHAPES’ Ecosystem and socio-technological innovations will be responsive to demographic, local, and national variations. Furthermore, findings about, for example, differences in internet access and digital connectedness, gender inequalities, governance structures, health and care system accessibility, and urban and rural divisions of older adults, will be considered in building the SHAPES Platform as an adaptable and flexible system. Importantly, SHAPES will not merely set short-term objectives for the life cycle of the project. Instead, it will focus on the long-term impact the project can have on the future of European health and care systems. SHAPES proposes to validate cost-effective, interoperable, and reliable innovations capable of effectively supporting healthy and independent living of older individuals within and outside the home. In particular, the SHAPES Marketplace seeks to connect demand and supply across H&C delivery, and to facilitate the co-creation of affordable, effective, and trustworthy solutions. A catalogue of solutions and services facilitates the transparent expansion of the market offer, prevents vendor lock, and enhances the competitiveness of the EU health and care industry. Finally, the project aims to develop value-based business models to open and scale up the market for digital technologies supporting active and healthy ageing. Moreover, to provide policy recommendations for the successful implementation of innovative digital health and care solutions and services for older adults in and beyond Europe. Specifically, SHAPES will provide guidelines, a roadmap, and an action plan, including a set of priorities dedicated to standardisation, to support key EU stakeholders to foster the large-scale deployment and adoption of digital technologies and new integrated care services in Europe. 

### Responding to the SARS-Cov-2 pandemic

While it is important to note that the SHAPES objectives were designed in a pre-pandemic socio-political, economic and care context, the project will operate during the COVID-19 pandemic. The pandemic has disproportionately affected older adults, and it is globally accepted that people in the later stages of their lives are at a higher risk of serious morbidity and mortality associated with COVID-19 ( Brooke & Jackson, 2020). It is estimated that COVID-19 infection rates exceeding 1% to 2% of the population are likely to lead to rapid reduction in life expectancy in all EU Member States where life expectancy was previously high ( Marois *et al.*, 2020). The pandemic prompted changes to planned research activities. Instead of visiting both older people in their homes and the pilot sites, partners will largely gather data remotely. Naturally, this entails methodological changes, adaptations, and certain limitations, such as reliance on participants’ detailed descriptions of their environments. Despite the challenges, the situation might also bring about insights resulting that may guide the development of the Platform, the digital technologies and the piloting activities. The pandemic has accidentally created the ‘real world’ context within which older adults sheltering in place were compelled by circumstances to adopt technologies to enable them to remain in touch with family, friends, and care providers. In addition to the adapted approaches to data collection, this altered reality will be acknowledged in SHAPES through the development of digital technologies for the monitoring and measurement of symptoms, and support of patients in quarantine. Going forward, SHAPES’ research activities will continue to adapt in response to the pandemic.

## Structure of the project/work packages

**Figure 3.**
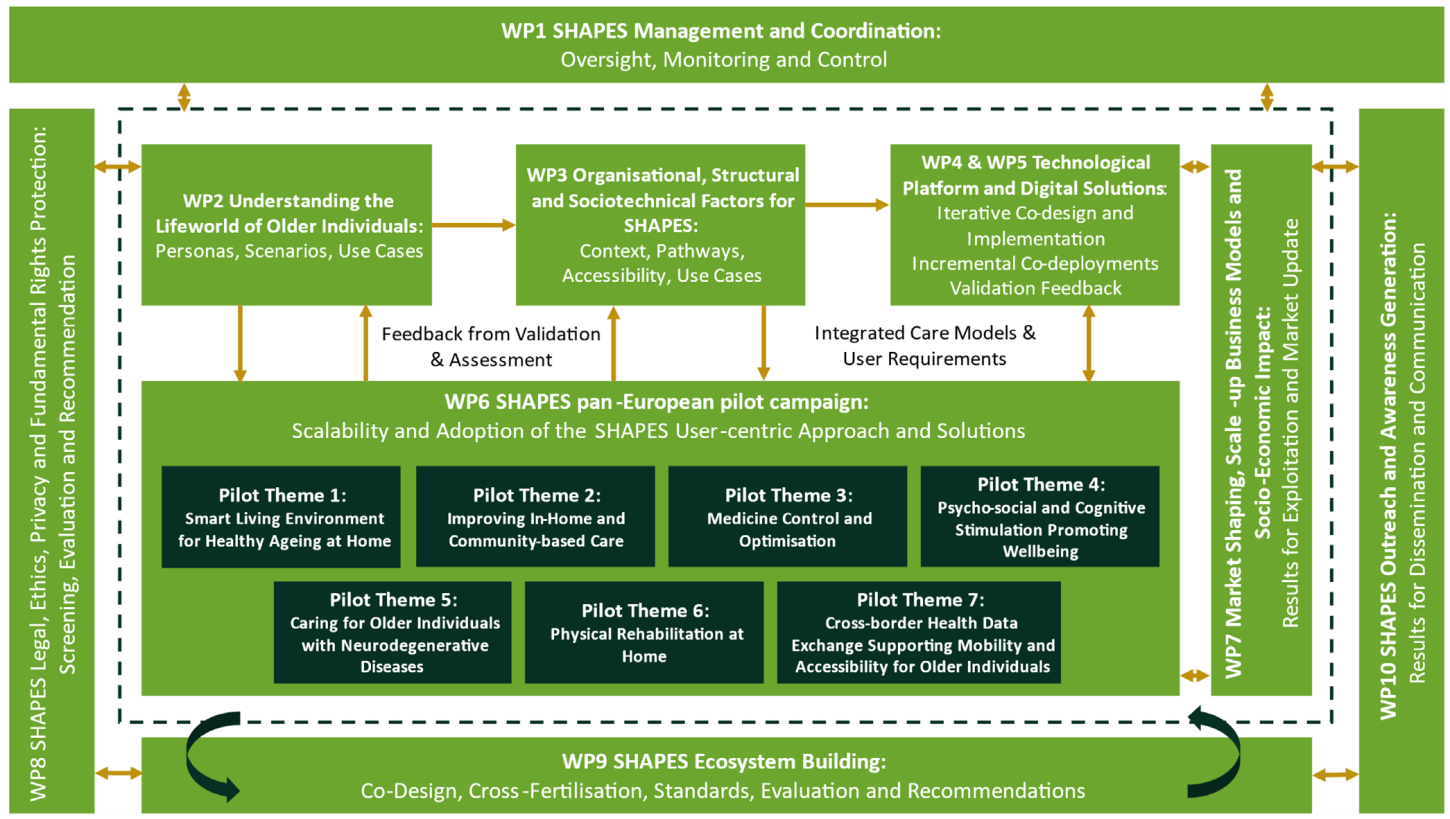
Overview on the development of the SHAPES Platform and Ecosystem.

As illustrated in Figure 3, the development of the SHAPES Platform and Ecosystem will involve a substantial amount of groundwork across multiple work packages (WPs), including empirical studies (WP2 and WP3) and the development and implementation of the Technological Platform (WP4) and Digital Solutions (WP5). These activities support the Pan-European Pilot Campaign (WP6), which seeks to validate the SHAPES Platform. All activities contributing to the development, implementation, and validation of the Platform take place within ethical and legal frameworks and are supervised and guided by internal and external ethical and legal experts (WP8). The findings from research, development, and pilots inform further activities concerned with market strategy and business models for SHAPES (WP7) and the SHAPES governance model and Ecosystem that build on the principles of openness, interoperability, expandability, and modularity (WP3, WP4). Concurrent efforts involve activities to build the SHAPES ecosystem (WP9) and dissemination (WP10). The following sections describe the range of SHAPES activities more fully.

**Figure 4.**
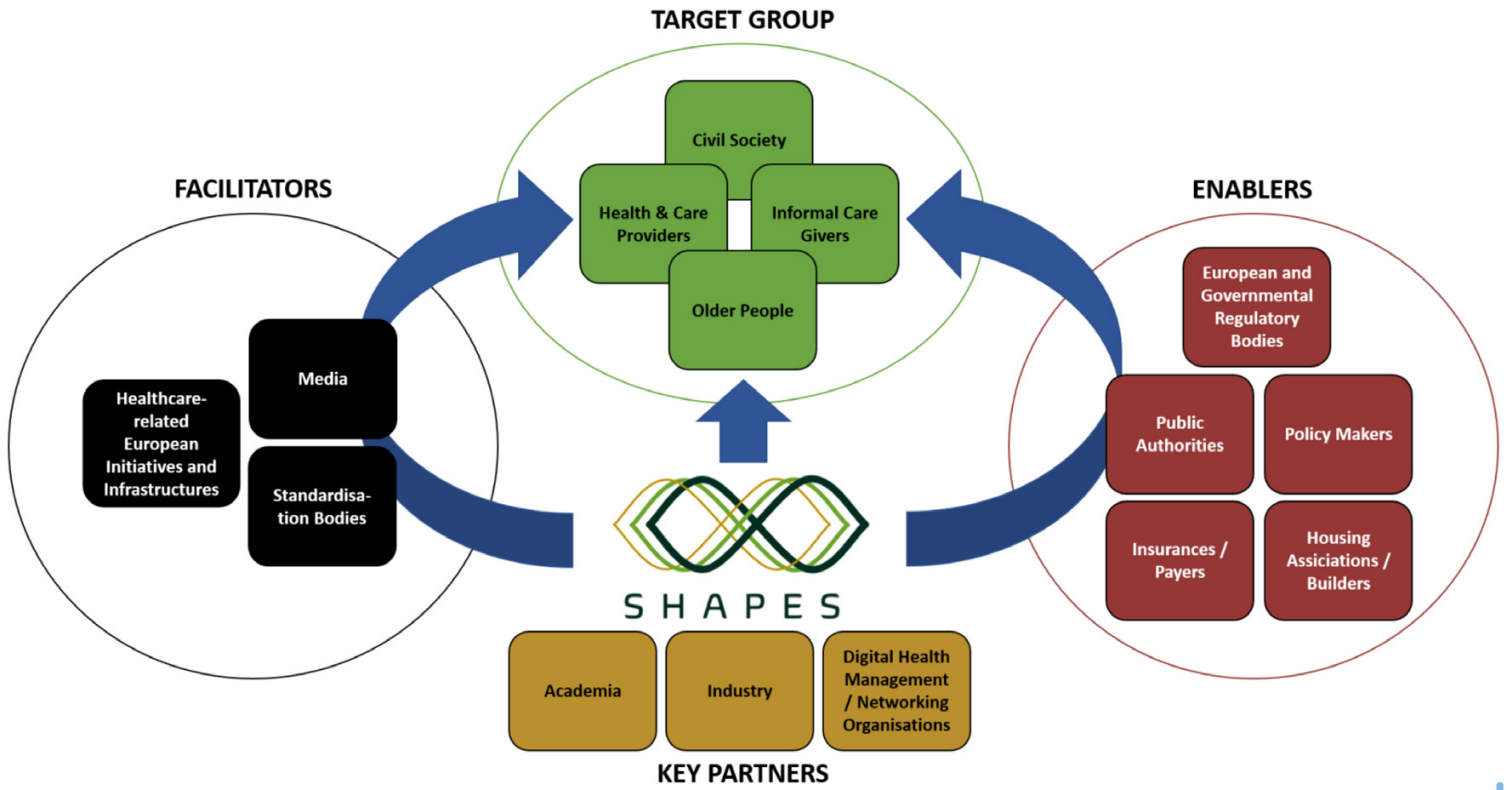
Overview of SHAPES ecosystem stakeholders.

### Understanding older adults, their lifeworld, and social contexts

The activities in both WP2 and WP3 will be essential for the development and implementation of the SHAPES Platform. WP2 explores ethnographically the experiences and expectations of ageing from the perspectives of older people and their networks across Europe. The goal of this work package is to gain a detailed understanding of individuals, their environments and real-life contexts of ageing in different pilot sites across Europe. The results from this research are intended to be both locally useful and available for cross-European comparison. The insights that will emerge from the empirical data explore relationships, neighbourhoods, and environments. Moreover, ethnographically informed qualitative data from case studies with older adults who live in different European countries who have various health and care needs will guide the iterative co-design of the SHAPES Platform, the selection of appropriate care interventions, and the evaluation of both SHAPES innovations and care environments. This understanding of lifeworlds in the context of networks of intimates, communities and care ecologies is the ethos of the SHAPES Ecosystem, making it culturally responsive to stakeholders, including older adults, informal caregivers, formal healthcare providers, community groups and civil society (see Figure 4). The detailed and comparable picture of older adults’ way of living, and their perceptions of ageing, their care networks and their reflections on formal and informal health and social care sectors will be translated into personas and use cases. These constitute a baseline for the SHAPES Platform, for the selection of appropriate socio-technical interventions and for subsequent evaluations. Insights pertaining to these lifeworlds complements the research in WP3, which will explore the organisational, structural and sociotechnical factors for the SHAPES Ecosystem, and the development of the SHAPES governance model. These various perspectives facilitate the development of participatory processes for meaningful empowerment and engagement of older individuals in the definition and implementation of active and healthy ageing and independent living policies.

### Organisational, structural and sociotechnical factors for the SHAPES ecosystem

As stated, complementary to the activities in WP2, WP3 will explore the sociotechnical and organisational systems within which health and social care delivery takes place across Europe. Prior to the SHAPES pilots (see below), WP3 will examine the organisational and socio-technical aspects that govern health and care systems. Seeking to develop a future-orientated concept of operations (CONOPS) ( Institute of Electrical and Electronics Engineers, 2007), underpinned by cultural historical activity theory ( Engeström, 1987), this work package pays particular attention to the human factors, i.e. individual, managerial, cultural, and environmental aspects that facilitate or hinder care delivery. In addition, based on both use cases and the evolving CONOPS, this baseline work in SHAPES will provide an exhaustive description of diverse user requirements, such as technical, functional, legal and ethical, security and business requirements, that guide the design of the Platform in an iterative cycle of feedback and refinement. Simultaneously, activities will take place to analyse best practice examples of existing integrated care programmes to establish ways of scaling up technology-mediated integrated care delivery. Furthermore, these tasks will result in policy-oriented guidelines for the implementation of technology-mediated integrated care models across Europe. Moreover, in-depth analyses of various aspects of governance (care, IT, legal and business), in collaboration with various work packages, (WP2, WP7, WP8 and WP9), will result in the SHAPES Governance Model and Guidelines for the Platform. In tandem, WP2 and WP3 are providing a comprehensive picture of the reality of health and social care delivery in Europe from both an individual and systemic perspective. Crucially, the activities in both work packages are fruitfully situated to capture the socio-cultural, psychological, and economic dimensions of health and social care systems resulting in the contextual backdrop for the SHAPES Platform and WP6, the piloting activities.

### Building the SHAPES platform and digital technologies

WP4 and WP5, in continuous exchange with WP2, WP3, WP6 and WP8, will develop the technology for the SHAPES Platform that includes a suite of digital solutions. To be able to successfully mediate the delivery of health and social care, the Platform needs to be open, interoperable, expandable, and modular. Whether the platform can adequately fulfil these requirements will be evaluated during the piloting activities (WP6). The development of the Platform will take into consideration the functional, ethical and legal, security, health system, business and technical requirements identified in WP3, and the personas and use cases developed in WP2. Mapping these requirements to a reference architecture and to the implementation of the SHAPES technological platform (TP) and its core components will allow interconnection among the various SHAPES digital components and solutions. Integrating findings from WP2 and WP3, emphasis is given to core functional components, authentication, security elements, and interoperability services, in compliance with standards or widely adopted and emerging protocols, ensuring SHAPES’ openness, interoperability and security. Furthermore, WP4 will deal with and verify the integration of digital technologies (developed in WP5) in the SHAPES TP.

WP5 will tackle the definition of the SHAPES User Experience (UX) and provide guidelines for the implementation of digital technologies specifically, those designed for older users. Moreover, SHAPES digital technologies will be adapted to comply with user requirements and follow the SHAPES architecture, benefitting from software development kits (SDKs) and application programming interfaces (APIs) developed in WP4 and WP5. The digital technologies also benefit from SHAPES’ advanced features, including speech processing and natural language processing (evolving text-based chatbots capable of engaging in human-like conversation) and hardened security and privacy (using e.g., multimodal authentication).

### The SHAPES pilot campaign and evaluation activities

Piloting and evaluation of the SHAPES Platform and its (digital) technologies, both under controlled conditions and in real-world contexts, is essential. The SHAPES pan-European pilot campaign (WP6) will involve both small-scale and large-scale pilots in eleven European countries (Czech Republic, Cyprus, Finland, Germany, Greece, Ireland, Italy, Portugal, Spain, Sweden and the UK). The pilot campaign is structured around seven themes that focus on improving the health, wellbeing, independence, and autonomy of older individuals, while concurrently enhancing the long-term sustainability of European health and social care systems. It will involve older adult end-users, caregiver end-users, and care professional end-users in 25 different use cases, which will conduct their pilot activities in 15 pilot sites, including five EIP on AHA Reference Sites. The spectrum of pilot themes enables the validation of innovations that capably support, promote, and extend active and healthy ageing, and independent living of older adults who have permanently or temporarily reduced functionality and capabilities within and outside their homes, and does so in a cost-efficient, interoperable and reliable manner. To understand whether and how SHAPES might meet the needs of its end-users, the pilot campaign is founded upon the knowledge base developed in other SHAPES work packages, including the identification of SHAPES personas and use cases (WP2), understanding existing care pathways and care delivery (WP3), and the development of the SHAPES Technological Platform and solutions (WP4, WP5).

The SHAPES pilot campaign will be implemented in a series of phases, which altogether will include 2,000 participants. Phase 1 will involve planning of pilot activities, pilot design in consideration of end-user needs (user requirements, scenario, data plan), and the specification of key performance indicators (KPIs). This phase will draw upon the detailed understanding of older adults and their lifeworlds developed within WP2 of SHAPES *.* For Phase 2, SHAPES technical partners will have provided mock-ups for each use case. At this stage, participants in the pilot activities will be invited to give feed-back to be able to design the SHAPES digital solutions tailor-made to the needs of the end-users. In Phase 3, the digital solutions will have reached prototype stage. Participants will be asked to provide a second round of feed-back. Additionally, this phase will include hands-on experimentation to train the participants on the individual digital solutions. Phase 4 will involve small-scale live demonstrations, where a small number of participants will be asked to replicate the scenarios described in Phase 1. Phase 4 will act as a ‘play rehearsal’ seeking to ensure that specific combinations of different SHAPES digital solutions integrated into the SHAPES Platform work as planned in participants’ homes environments. In Phase 5, the piloting activities will be carried out in real life with approximately 450 participants. This phase will involve the testing of the platform and its digital devices in participants’ home environments, as described in Phase 1. Each pilot theme will be replicated in several pilot sites. Different pilot activities are associated with particular themes, and they are conducted by one leading pilot site (L) and replicated, in part or whole, by other pilot sites (R). Each pilot site may conduct different pilot activities addressing different themes depending on their relevance to the end-users.

SHAPES will encourage the trialling of third-party technologies provided by organisations from outside the consortium, and their inclusion of external companies will be facilitated through open calls. This will contribute to both the expansion of the platform across Europe, and to evidence-based decision-making processes pertaining to the adoption and market uptake of SHAPES policies and digital technologies for active and healthy ageing (AHA).

### Shaping the market

The long-term sustainability of the SHAPES is a key concern that is reflected in the high-level adoption and expansion of the SHAPES Platform and its digital solutions in the post-project phase. WP7 will define strategies to manage – i.e. shape - the market, which will inform the business models developed in SHAPES to maximise returns within the parameters of a healthy market. WP7 will develop strategies to elicit the best value for money for both users and customers, and for the internationalisation and global ambitions of SHAPES. A SHAPES ‘marketplace’ will be developed to explore the Platform’s modular architecture, enabling the establishment of a rich ecosystem of suppliers and functionalities, and obtaining a significant market advantage.

The silver economy encompasses economic activity centred on the needs of people aged 50 and over, and includes an innovation and technology as a central component. It is expected that by 2025, it will generate €6.4 trillion (31.5%) of the EU’s GDP and over 88 million (37.8%) jobs . It is also expected that healthcare spending will rise from 53% to 60%, which will account for €465 billion (11.4%) of all government spending ( European Commission. Directorate General for Communications Networks, Content and Technology *et al.*, 2018). To support and sustain SHAPES’ integrated care vision in the long-term, WP7 addresses the silver economy and active and healthy ageing market idiosyncrasies, economic drivers and business models, and economically efficient funding models.

The digital solutions in SHAPES will touch several markets in the silver economy, including connected integrated care services and health platforms, robotics, gaming and mindfulness apps, intelligent living platforms, and health-related silver tourism. The SHAPES Platform will offer a set of sustainable technology-based solutions that will produce a significant body of knowledge and evidence that further promotes the opportunities for wellbeing in the later stages of life. The SHAPES Marketplace will build on this approach, providing reliable and trustworthy knowledge as a valued tool to support health and wellbeing and creating an interactive, dynamic platform connecting the demand and supply sides. It will function as a *one-stop-shop* that delivers a dynamic catalogue of solutions and services and will foster the transparent, pan-European expansion of the market offer, prevent vendor lock, and enhance the transparent competitiveness of the health and care industry in Europe. The SHAPES Marketplace also incorporates informational resources, best practices, tutorials and educational material, aiming to nurture the community in general and the H&C service providers in particular, with respect to the most optimal settings, models and policies involving integrated care in Europe and the process of ageing in place.

### Ethical and legal dimensions of SHAPES

Ethical and legal dimensions are a crucial part of SHAPES. WP8 aims to ensure that ethical innovation and development standards are met through the definition of ethical requirements for the Platform, identification of ethical and legal concerns regarding data protection and privacy as they relate to the Platform, and through the development of a regulatory framework for SHAPES. It further ensures that SHAPES acts in an ethically responsible manner so that it can be regarded as a positive innovation for its various end-users and service providers. This means that ethics is an opportunity to create more value, not only a source of risks.

The work package complements the other WPs by locating the SHAPES Platform and SHAPES digital technologies in the EU ethical, legal and policy frameworks. All SHAPES activities must comply with relevant legislation at EU and national level. These include the Charter of Fundamental Rights of the European Union, and relevant EU legislative acts such as the Directive 2011/24/EU on patients’ rights in cross-border healthcare and General Data Protection Regulation (GDPR). In addressing the needs of older adults with disabilities, SHAPES activities are also carried out in compliance with the principles and the rights provided for in the United Nations Convention on the Rights of Persons with Disabilities (2006). To ensure the requirements and perspectives of older adults with disabilities are included in the project, expert partners in disability (EUD and WFDB) are continuously consulted, including on matters related to physical and digital accessibility, common barriers, recommendations and good practices to inclusion. Additional theoretical frameworks support a person-centred deployment of technologies, such as biomedical ethics, the ethics of care, and the capabilities approach, which establish the value base for the WP8 work, and the SHAPES Ethics Framework. WP8 takes into account that the EU legal landscape on digital technologies is evolving and investigates ongoing policies as well as recent legal developments relevant to the project, including and not limited to the recent proposals for a Digital Services Act, a Digital Markets Act and a Declaration on digital rights and principles ( European Commission, 2021).

### Ethical compliance across work packages

SHAPES hosts an Ethical Advisory Board to provide relevant guidance to the project. The SHAPES Platform considers the relevant H&C delivery landscape to address the integrated care models and policies in Europe and the ageing in place process, as well as EU approaches to harmonise eHealth, assistive technologies, and the cross-border exchange of healthcare information. Since EU legislation takes the form of minimum standard directives, national implementation plays an important role. Within the Consortium, 13 EU Member States and Norway are represented, thus providing a wide regulatory coverage for the implementation of the SHAPES Platform. SHAPES identifies the most relevant regulatory specifications applicable to H&C information exchange cooperation, including in the context of prevailing H&C models and systems, considering personal data exchange (e.g., data retention rules, ownership, storage, access, necessary intergovernmental agreements). Further, SHAPES dedicates specific attention to EU standardisation efforts on eHealth and to the security risks and challenges related to the design and development of the SHAPES Platform and digital technologies.

The knowledge gathered through the multitude of tasks and work packages of the SHAPES project will yield practical results, including i) a handbook detailing the methodology for organisational implementation of the SHAPES Platform and ii) a report capturing the key recommendations for the implementation, adoption, and scale-up of the SHAPES Platform and digital technologies across Europe.

### Ecosystem building

WP9 focuses on building an interactive ecosystem that comprises a heterogeneous set of stakeholders on both multidisciplinary and international level spreading over different European health and social care systems. In parallel to forming its platform, pilots and digital solutions, which are all interconnected with understanding the lifeworld of older people and substantial ethical requirements, SHAPES aims to ensure sustainability for its actions beyond the project’s runtime. The basis for this sustainability is for SHAPES to understand the ecosystem in which it operates and its key stakeholders to provide assistive services and recommendations to help facilitate active and healthy ageing. The acquired knowledge will enable SHAPES to actively form and build its ecosystem of reference to ensure uptake and long-term integration of the project outputs.

In particular, WP9 aims to achieve this goal through three core activities: Running a Co-creation Think-tank for European Integrated Care that comprises internal and external members to discuss governance and data management emerging from WP3. Performing Innovation Watch, Cross-Fertilisation and Foresight Exercises to identify emerging technologies, future impacts and new societal demands and challenges that help to evaluate priorities and potential new directions in decision making. And building strong networks and liaisons to analyse, adapt and recommend standards and interoperability and to manage Open Calls for Innovation and Collaboration that will give external organisations the chance to participate in SHAPES and to enrich the portfolio of SHAPES digital solutions. The gathered information is summarised in “Influencing Factor Cards” and “Technology Cards”, that provide starting points for interested actors for more in-depth analysis into specific topics.

### Outreach

To support the set-up of the SHAPES ecosystem, WP10 conceives seven dialogue workshops that engage external stakeholders in the project activities and are dedicated public frames where SHAPES meets a wide audience of researchers and businesses, health and social care professionals, older people and civil society. Each workshop is anticipated by an awareness campaign, circulating some key research questions of each event to its social media audience. Through the awareness campaigns, SHAPES reaches out to an informed public and confronts with it, exchanging on concepts, methodologies, outputs and results, to further deepen the project’s knowledge and outreach.

## The panoply of methodologies adopted in SHAPES

SHAPES implements a co-creation methodology between the SHAPES partners and the SHAPES stakeholder ecosystem and between the Action’s social science and technological development. This occurs in six-month cycles, each delivering results and outcomes that take the Action a step closer to achieving its strategic goals.

The technology developed and adapted in WPs 4 and 5, and validated in WP6, will be informed by user needs, priorities, and requirements (WPs 2, 3 and 9) and is in full compliance with EU and national legal, ethical, and fundamental rights framings (WP8), in which the General Data Protection Regulation and the Directive 2011/24/EU on patients' rights in cross-border healthcare are key pillars. SHAPES adopts an ethics-based approach taking the protection of the rights of older individuals as central. 

### The SHAPES ethnographic research methods

The social science activities in SHAPES combine engagement in knowledge exchange with existing projects and mini ethnographic studies, lending itself to both comparability (between case-countries) and customisation (tailor-made approaches per country). 100 ethnographic case studies to understand the interconnected, contextualised, sociocultural and gendered lifeworlds of older individuals and persona-based use cases to investigate the meso and micro levels enable an exploration of differences and similarities to build local-context-specific knowledge. In-depth open-ended ethnographic interviews and participant observation, expert interviews and focus groups, and intervention-based methods are applied. Thereby, the research considers obligations emanating from relevant law, ethics and policy and provides the relevant requirements to assist the development of the technological solutions.

### The SHAPES activity-centred concept of operations

SHAPES will develop an activity-centred concept of operations (CONOPS), which is based on the IEEE’s standard CONOPS template ( Institute of Electrical and Electronics Engineers, 2007) and enriched with Engeström’s (1987) activity system framework. A concept of operations is a future-orientated representation of an operational system that incorporates the proposed innovation. Its main purpose is to promote dialogue around its direction and development. A good CONOPS is one that allows each stakeholder to see themselves in that operational picture and be informed about how they might likely be impacted by it. The CONOPS approach enables an understanding of the current ‘as is’ situation pertaining to health and care systems in terms of their human, organisational, material, and informational elements, and the relative roles of each in achieving the system outcomes. Furthermore, it provides a framework for analysing the strengths, shortcomings, and gaps in the current system. Moreover, through focusing on opportunities for improvement through innovation, it acts as a guide for the development of the SHAPES Platform, taking account of existing structures, processes, and human factors. 

The activity-centred approach to CONOPS emphasises the interconnectedness of different actors with a focus on both their shared and diverging goals and interests with a broader sociocultural and material context. This framework complements the CONOPS framework as it views the developing technological ecosystem of SHAPES as a key mediator between people and their goals and allows the SHAPES partners to view the development of the Platform with a constant eye of the intended future state. Rather than trying to ignore or avoid potential conflicts in the system, which may occur due to divergence between the goals and interests of actors, the availability and suitability of resources, and the challenges surrounding organisational change, it seeks to proactively identify such potential contradictions and position them as key design challenges. The purpose is not to eliminate all potential conflict but to develop a sociotechnical system with the flexibility and inclusiveness required to accommodate the range of diverse actors and goals as is reasonably possible. 

### The use case co-creation method

To understand and analyse end-user needs, and ultimately evaluate whether SHAPES meets those needs, the project employs a human-centred, stepwise, co-design process by developing personas, user stories, use cases and technologies. Firstly, in WP2, social scientists and partners within the SHAPES consortium produce personas from ethnographic case studies and focus groups with older adults and caregivers as well as in-depth interviews with professionals. Personas will contain the attributes, attitudes, behaviours, and characteristics of the various end-user groups targeted by SHAPES. Secondly, in WP2, general use cases (UCs) are developed on the basis of the needs of personas. Use cases aim to illustrate the breadth and variability of the technology use for improvements of the quality of life of older adults. Lastly, in WP6, on the basis of the more general use cases developed in WP2, specific use cases are developed for each of the pilot themes (UC-PTs). In these UC-PTs, the needs of end-users and the user stories are merged with specific digital tools and solutions of the SHAPES Platform.

### Technological development and deployment methods

For the technological development and deployment activities, SHAPES applies a three-stage methodology: 1) Prototyping – to consider the end-users requirements and deliver hands-on experience of mock-ups for early validation by end-users; 2) Development – design, implementation, adaptation and integration of the Platform, improving existing solutions; and 3) Deployment – installation of the Platform and digital technologies in users’ real-life environment.

**Figure 5.**
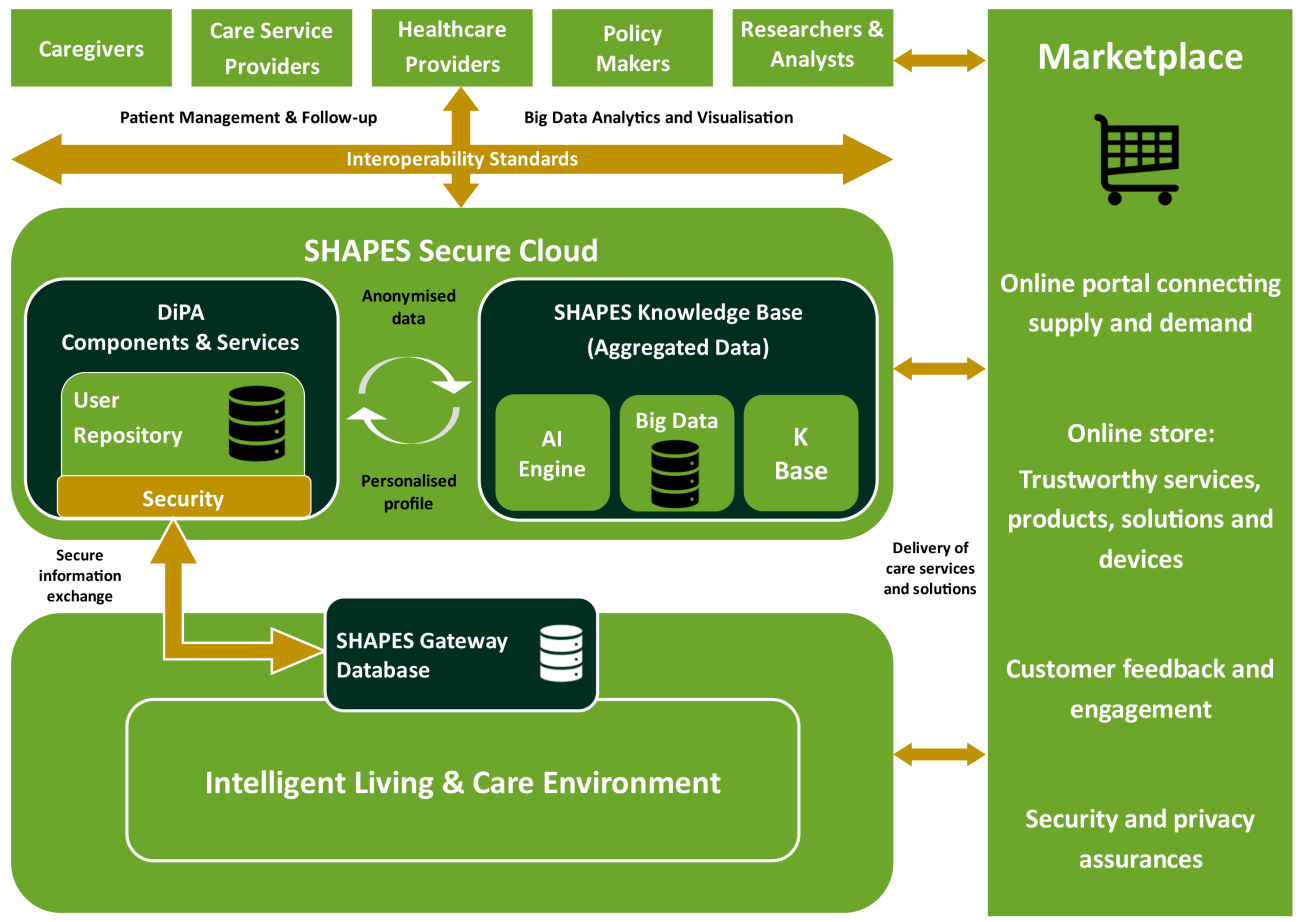
SHAPES High Level Architecture.

In the background of the SHAPES architecture ( Figure 5) lies the “Intelligent Living and Care Environment”, a collection of services, devices, applications and other types of solutions that offer e-Healthcare support to diverse classes of stakeholders for a broad range of needs. As most of those are developed independently and not always compliant with common interoperability criteria, their capabilities to communicate and exchange data and information may be limited. This is where the SHAPES architecture comes into play, offering interoperability mechanisms as well as additional capabilities (e.g., AI-based analytics) with advanced security mechanisms, thus enabling seamless integration of originally dispersed and disconnected services and applications into collections of tools for addressing similar needs and leveraging their individual advantages to mutual added benefit.

Through the SHAPES concept of a Marketplace, such extended new types of solutions can be advertised, discovered and consumed by stakeholders as well as combined together to address new and emerging e-Heath needs, an example being the current COVID-19 pandemic that has identified a need for new types of e-Health solutions targeting large–scale infectious diseases. To offer technological capabilities that address such generic user needs, a practical implementation of the Technological Platform differentiates device, middleware and service layers at the bottom of the diagram that are all associated with symbIoTe platform as the core IoT interoperability platform. Using its multiple levels of interoperability compliance, devices, platforms, and services are seamlessly and flexibly registered to symbIoTe, match their individual data information models to produce conversion rules, allowing them to directly and bilaterally exchange their information. This way privacy of data can be achieved compliant with GDPR regulations. Such a mediation framework offered by symbIoTe further permits flexible addition of new types of solutions thus supporting expandability of the SHAPES system to new solutions in the future.

Using 3rd-party expandability capabilities using concepts of “enablers”, representing approaches ranging from software development kits (SDKs) and application programming interfaces (APIs) to explicit reusable code offered as Docker components, new solutions, services and applications can be grown on top of the SHAPES technological platform to address new and emerging e-Health domains. The core architecture defines the underlying system organisation and its components whose purpose is to support seamless connectivity and data exchange/sharing among interconnected devices, services, platforms and applications (as described in the concept architecture earlier), all of which being referred to as Digital Solutions. Such an architecture has been defined to offer the following basic functionalities:

1. Single sign-on to all Digital Solutions compatible and registered to SHAPES

2. Data authorisation managed by users and on Digital Solutions where data is stored

3. Seamless integration of devices, services, platforms and applications

4. Standard-independent interoperability w.r.t IoT data and Medical Information

5. Advanced data analytics of data shared among diverse Digital Solutions

6. Management of medical data over diverse Digital Solutions for same individual

7. GDPR-grade protection of private identifiable private data

8. Future extendibility to additional medical standards and data models

9. Optional sharing of captured anonymised data sets for research purposes

SHAPES will deploy these digital solutions in the homes and communities of end-users of the Platform. Table-top exercises, implemented at the early stages of the Action, will validate initial concepts and approaches, by recreating realistic scenarios. Mock-up or prototype validation, performed at the early stages of the Action, will assess user acceptance and feedback on user experience and other non-functional elements, such as accessibility and inclusion features. Hands-on experiments, conducted at the mid and late stages of the Action will validate functional elements of SHAPES components and selected digital technologies and gather user feedback on performance, shortcomings and new additions. Based on the feedback, the digital technologies will be adapted and improved and be tested again by the older adults.

### Evaluation methods

The SHAPES Platform must be validated at the European scale, across different regions, cultures and health and care organisational models and on the basis of its overall aims. Evaluation will be facilitated by a range of both quantitative and qualitative methods and analyses and, rather than focusing merely on individual technologies or themes, SHAPES will be evaluated as a platform and ecosystem, and as a comprehensive set of potential solutions to a set of complex issues. Care technologies must meet the needs of their target beneficiaries, and do so effectively.

For each use case, key indicators relating to health, quality of life, and wellbeing, will be developed and the constituent technologies, will be evaluated in relation to their impact in such indicators. As the ecosystem aims to be greater than the sum of its constituent parts, in addition to data collection for assessment of outcomes of individual pilots, a core set of harmonised psychosocial data will be collected across the pilots. This core set of data is also used together with additional use case specific data to evaluate the outcome of each use case with MAST. MAST is an established method to assess the effectiveness and contribution of telemedicine applications to quality of care ( Kidholm *et al.*, 2012; Kidholm *et al.*, 2017). MAST defines the assessment as a multidisciplinary process that summarises and evaluates information about the medical, social, economic and ethical issues related to the use of telemedicine.

For the overall evaluation of the SHAPES project, SHAPES intends not merely to undertake comparative evaluation of individual use cases, but to evaluate SHAPES at the level of the Platform, and in holistic terms. SHAPES will also be evaluated for its gender transformation capabilities (changing the power relations that underlie inequalities), incorporating gender sensitivity (describing inequalities), and gender specificity (taking action to compensate for inequalities). The promotion of health and quality of life is bound by politico-economic and fiscal imperatives, and socio-economic sustainability and cost-benefit analyses will be performed within SHAPES.

Health technologies, although they may be promising, are often not adopted by end-users to a substantial degree, are not accepted by end-users, are not scaled-up within organisations or contexts, do not spread to other organisations and contexts, and are not sustainable ( Greenhalgh *et al.*, 2017). Complexity is often unaccounted for and is at the root of many such failures. Complexity may be associated with the technology, relevant health conditions, value proposition, adopters, organisation, wider systems, or embedding over time. This complexity and risk of such failures will be considered and planned for within SHAPES.

Thus, prior to the beginning of the pilot activities each use cases runs through a set of tests. First, the use cases are checked by using the NASS framework Greenhalgh *et al.*, 2017). The NASSS framework was formulated to explain the high number of failed technology projects in health care. By using the NASSS framework a research project is analysed in seven domains (condition/illness, technology, value proposition, actual or indented adopters, organisation, wider system, process of adaptation) and for each domain it is decided, if the project plan is simple, complicated or complex. If in several domains the project plan is complex, the project has only very limited chance of being successfully implemented. The aim is to detect, if the use case and the related planning of the pilot activities are in some ways too complex and have to be adapted.

Additionally, the use cases are checked with the MOMENTUM (2014) method, which identifies critical success factors needed to take telemedicine from the pilot phase to large-scale deployment and thus aiming to integrate it into the healthcare delivery system. Overall it contains 18 critical success factors in the condition areas ‘strategy and management’, ‘organisation and management’, ‘legal and security’ and ‘technology and market’. The degree to which these critical success factors are fulfilled indicates the extent to which the organisation/region is ready for large-scale deployment of the respective telemedicine solution (or use cases in the case of SHAPES).

SHAPES will define and implement a user acceptance, inclusion, and societal impact assessment model. This work will reinforce and validate compliance of the SHAPES Platform to the identified user and ethical requirements and assist in risk detection and prevention, and the adoption of tailored safeguards to ensure the societal acceptance of the SHAPES Platform and digital technologies.

As a whole, SHAPES and whether or not pilots advance toward the use case objectives, will be evaluated with a range of KPIs that are measurable and quantifiable and have target or threshold values. To evaluate the SHAPES Platform and vision in holistic terms, a core set of harmonised psychosocial outcome data will be collected from participants across the pilots at pilot baseline, end-of-pilot, and a three-month follow up. Psychosocial assessment will include, but not be limited to, quality of life, health-related quality of life, self-efficacy, social support, participation, health literacy, system usability, and technology acceptance. This data may also permit analyses by the SHAPES Data Analytics Engine techniques. 

### Market shaping to inform SHAPES’ business models and socio-economic impact

To enable older people’s autonomy and active, healthy lives, SHAPES seeks to achieve equitable health and social care delivery through availability, accessibility, and affordability, as well as economic sustainability, competition and innovation locally, nationally, at European level, and internationally. SHAPES will contribute to both the improvement of older people’s health and quality of life, and to the sustainability and efficiency of care systems and create market opportunities for businesses and industry.

Achieving these goals depends on a well-functioning market. However, incongruities that exist between demand and supply, quality and affordability, as well as the relationship between public and private sector procurers, producers, and suppliers ( MacLachlan *et al.*, 2018) can result in a breakdown of the markets for assistive devices. Therefore, it may be necessary to manage **—**or ‘shape’ **—**the market, meaning to establish “strategic relationships between public and private entities, through for example pooled procurement or development and distribution risk sharing, at regional, national, and international levels” ( MacLachlan *et al.*, 2018, p. 3).

SHAPES will deploy a market shaping strategy to facilitate equitable access to health and social care for all, utilizing a range of mechanisms such as the SHAPES Marketplace to connect supply and demand. The Platform aims to increase access to traditionally smaller niche markets, allowing for economies of scale, reduced pricing, the provision of product maintenance, or user training courses, virtual or physical. The SHAPES market shaping strategy will endeavour to make the SHAPES Platform attractive to industry and policymakers. This will be done through a) optimisation of a growing SHAPES ecosystem, b) building of critical mass, c) cost reduction through the expansion of integrated technology-mediated care delivery, which may lead to greater market competitiveness, d) accelerated transfer of knowledge and technologies, and e) facilitating access to non-EU markets. Key to achieving this is the delivery of new and innovative digital solutions and services.

SHAPES will take a system-of-systems ecological approach, which facilitates the analysis of systems as a network of interrelated relationships rather than siloed units. This approach recognises the role of decentralised services and local structures in accessing innovative products and services. It is expected to result in greater autonomy at local level emphasising the role of community-based services, and to strengthen the agency of service users.

**Figure 6.**
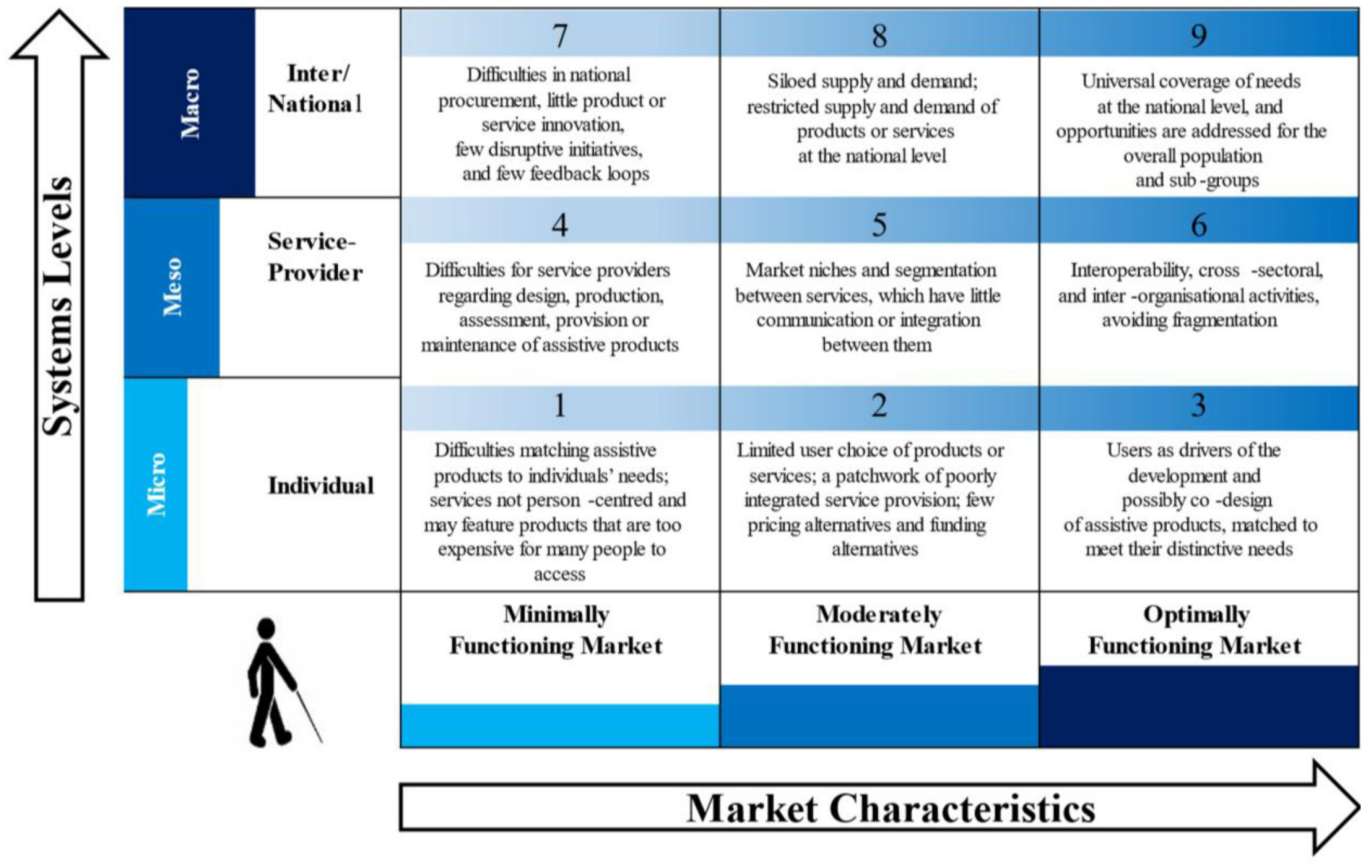
Systems Market for Assistive and Related Technologies (SMART) Thinking Matrix. Source: MacLachlan *et al.*, 2018, p.7.

SHAPES will utilise the *Systems Market for Assistive and Related Technologies (SMART) Thinking Matrix tool* ( MacLachlan *et al.*, 2018) ( Figure 6), which was developed by Maynooth University to describe how SHAPES will function in a minimal, moderate or optimally functioning market, with respect to individuals, service providers and social policy.

The SHAPES market shaping strategy will include the development of a market analysis, which will identify key success factors (KSF) for the SHAPES Platform and its digital solutions to ensure their competitive advantage. The market analysis will investigate European and global markets of digital technologies that support active and healthy ageing and facilitate independent living. This analysis will draw upon the business expertise within SHAPES, supported by the wider ecosystem of SHAPES stakeholders, and will take into consideration tools and models such as SWOT and PESTEL, the KSFs and prevailing market barriers.

In addition, SHAPES will conduct an objective cost-benefit analysis of the daily living activities of its users and day-to-day integrated care delivery activities. Based on the outcomes of the SHAPES pilot campaign, a set of indicators for the socio-economic sustainability of the SHAPES results will be defined. Highlighting the importance of balancing social, economic and environmental objectives, these indicators are intended to be used to assess the impact of the SHAPES adoption and scaling-up initiatives. Including forecasts for the next five to ten years, the SHAPES economic sustainability analysis will vary the SHAPES approaches (methods, tools, technologies, processes) to identify assumptions, bottlenecks, intellectual property aspects, economic outcomes and alternative development and funding strategies.

The analyses of both the market and economic sustainability of the project will inform the SHAPES business plan. This will include a roadmap outlining how the SHAPES Platform and its digital solutions in the global market (TRL9 ^1^ Platform) can be commercialised successfully to ensure that access to SHAPES is cost-effective, will maximise returns for SHAPES partners and will serve the needs of older adults and health and care systems. The SHAPES Uptake Roadmap, which will be informed by the SMART Thinking Matrix ( Figure 6), will provide estimated timeframes for SHAPES business exploitation, which will support SHAPES in its goal to reach the global market.

As a result, the SHAPES market shaping method, aided by the SMART Thinking Matrix tool, will facilitate a better understanding of relevant market aspects, economic drivers, and business models. This knowledge will present the foundation for recommendations on how to promote interest and encourage the early adoption of the SHAPES Platform and its digital solutions while being responsive to the needs of the ageing populations and providing better health and care delivery.

### SHAPES ethical & legal issues

Ethical issues in the SHAPES project includes both SHAPES development work (process), as well as defining ethical requirements as features of the proposed SHAPES solution during that development and implementation work (outcome, solution to be created). SHAPES pilots are multifaceted from the viewpoint of ethics, since they concern ethics of development work, validation of the ethical features of SHAPES and the use of SHAPES in real-time settings. In the pilots, validation activities will be conducted in an environment that expects the development version to be piloted itself, fulfilling the minimum legal requirements. Efficient and compliant management of the SHAPES data is required to increase the sustainability of project results and enhance the integrity and validity of the project methods. As the project progresses, any newly identified ethical issues will be addressed by mitigating options to the Consortium.

One of the key challenges for the SHAPES project is the need to balance users’ rights to privacy and data protection, while simultaneously allowing for the use of big data analytics to create user profiles and deliver personalised solutions feeding into the SHAPES Platform. The debate about balancing accessing and using personal data, in particular, health data and protecting users’ privacy rights has gained fresh momentum since the introduction of the GDPR and adoption of the European Commission’s Digital Strategy ( Custers & Uršič, 2016; Fears *et al.*, 2014; Mayer-Schonberger & Padova, 2015). However, the current legislative framework on informed consent, data protection and the re-use of personal data creates a national balance between the ability of digital providers to exploit research data to develop or improve technologies whilst protecting research participant’s privacy rights ( Chassang, 2017; Rumbold & Pierscionek, 2017). In terms of applied research projects, this balance must be sought in the design of effective DPIAs, which negate risks and ensure compliance with relevant GDPR, informed consent and other data protection requirements ( Chassang, 2017). In this context, SHAPES WP activities are designed to preserve and promote fundamental rights, including the fundamental right to privacy as protected by the Universal Declaration of Human Rights (Article 12), the European Convention of Human Rights (Article 8), and the European Charter of Fundamental Rights (Article 7). WP8 further outlines the key provisions of the General Data Protection Regulation 2019/679 as ethical requirements for the privacy and data protection, which must be observed by the SHAPES partners.

Throughout the lifetime of the project, all SHAPES partners commit to upholding ethical research standards, including the European Code of Conduct for research integrity. They are committed to delivering high-quality scientific outputs and to be transparent, ensuring deliverables’ reliability and impact. These features of deliverables are validated as part of the quality management procedures. The principles of maximising benefit and minimising harm, of social responsibility, of dignity of persons, of fundamental human rights and other issues mentioned in the Horizon 2020 ethical self-assessment are supported during the R&D work by taking into use ethical self-assessment procedure as part of the SHAPES governance structure. This ethical self-assessment is based on the Horizon 2020 template, but it is further modified for the specific purposes of the SHAPES ethics governance.

### Study status

At the time of submission of this protocol article, the project is halfway through its lifespan. Much of the groundwork – such as the ethnographic investigation of older adults’ lifeworlds, persona development and use cases and the analysis of the trends in health and social care provision across Europe for the evolving CONOPS – has been undertaken successfully. Results, which form the basis for the development of the platform, have been shared with all SHAPES partners. Ethical, technological, social and legal requirements for the SHAPES Platform have been developed as the groundwork for the implementation of the Platform. Furthermore, methodologies for testing pilot use cases have been developed, and digital solutions and necessary technological prerequisites have been created and cross-aligned among all partners. A suitable ethical framework has been established and is guiding the piloting activities. Moreover, we are in the process of developing the SHAPES governance model, which will address questions related to business, enterprise and data governance, and will be aligned with the newly launched European Health Data Space. Midway through the project, the focus is now on piloting activities. Other activities involve the development of the SHAPES ecosystem, its marketplace and think tank to ensure the lasting impact of the SHAPES project in the health and care sector.

SHAPES commenced only four months before the onset of the coronavirus pandemic and the work of the SHAPES consortium was considerably affected by Covid-19. The ways in which the consortium worked, both internally and in gathering first hand data, had to be adjusted and had to respond to the methodological challenges relating to lockdowns and the fact that the population at the heart of this research was at high risk from Covid-19. However, while the pandemic challenged all staff to work even harder to find solutions to continue the work and research in SHAPES, all deliverables have been submitted on schedule and with the highest quality.

### Dissemination of results

Throughout the lifespan of the project, SHAPES will engage in dissemination activities to communicate with academics, policymakers, the health and care sectors, and public audiences. Using social media and a website as well as a blog called #shapesstories, results from and the process of research within the Innovation Action are processed in a way to be both intellectually stimulating and easily accessible. SHAPES communicates its findings, including real life experiences of older adults and key questions that need answering in innovation research, at various conferences across Europe and beyond via paper presentations, blog entries and panel discussions as well as through accessible SHAPES dialogue workshops.

Publications are an integral part of the SHAPES dissemination strategy. A number of scientific articles in a range of open access and academic journals have been published to date, and many more are planned for dissemination over the next two years. Additionally, two manuscripts for books are in preparation: the edited volume “Rethinking Smart and Healthy Ageing in Europe“ (working title; publisher SpringerNature) and the monograph “From Legacies to Futures. Lifeworlds of Older Adults in Europe” (working title; publisher Berghahn).

The SHAPES platform as well as the SHAPES governance model, ecosystem, and marketplace will all provide opportunities to disseminate results and findings and to ensure access to SHAPES data and findings for future researchers and contribute to market shaping. Furthermore, through webinar activities, SHAPES engages with the European Parliament and with the WHO to foster its impact. Embedded in the Health and Care Cluster ^2^ established by the European Commission to connect the main Horizon2020 funded research projects, SHAPES, the work group on dissemination, fosters capacity building and the continuous collaboration and exchange with other leading researchers in the field.

## Discussion

In innovative ways, the “ *Smart and Healthy Ageing through People engaging in Supportive Systems*” (SHAPES) project will create the first European open Ecosystem that will enable the large-scale deployment of a broad range of digital technologies for supporting and extending healthy and independent living for older individuals. The Ecosystem will build on an interoperable Platform integrating smart digital technologies to identify older individuals’ needs and to provide personalised solutions that uphold the individual’s data protection and trust. The SHAPES Platform will offer a combination of devices, software, and accessible modes of interacting within the living environment that can adapt to the needs and priorities of older individuals, including those facing permanent or temporary reduced functionality and capabilities. Beyond the lifespan of the project, the SHAPES Platform will sustain increased efficiency gains in health and care delivery across Europe, bringing improved quality of life to older adults, their families, caregivers and care service providers.

Using an inclusive, action learning approach allows for the inclusion of bottom-up insights from empirical data collected in the ethnographic lifeworld research as well as the pilot site validation that will make the project innovative and responsive to the needs and discoveries during its four-year lifespan. Rather than imposing fixed research structures, the project partners will work closely and collaboratively with each other to ensure effective cross linking of all work packages and their associated activities.

The SHAPES Platform will be responsive to sociocultural and economic contexts and the users’ physical and mental states, therefore being able to adapt to their pressing needs and concerns and use nudges to encourage users to make smart, healthy and active decisions, as well as achieve improved levels of independence, active social participation and quality-of-life. In other words, the SHAPES Platform will drive interconnection and integration, physical activity, personal agency and in-person human and community relationships. In addition, the SHAPES Platform will bring effective and efficient mechanisms to augment social interactions between individuals and, where applicable and consented, caregivers and care and health service providers, with rich communications accessible to all (e.g., combined video, text and/or audio) and ancillary data (e.g., activity and vital signs) improving diagnosis, risk assessment and complications prediction to enable early intervention and avoid corrective actions.

As such, the SHAPES Platform will be intelligent, processing user data in real-time and adapting itself as a suite of resources and capabilities to flexibly provide the human, digital and mechanical wherewithal to improve a wide variety of real-life environments. Powered by a big data and artificial intelligence (AI) engine, the Platform will incorporate an understanding of the evolving environment in which older individuals actually live and iteratively follows-up and evolves (i.e., learns) based on how the resources are being used. Building an ecosystem attractive to European industry and policy-makers, SHAPES develops value-based business models to open and scale up the market for digital technologies focused on active and healthy ageing and provides key recommendations for the far-reaching deployment of innovative digital health and care solutions and services supporting and extending healthy and independent living of older adults in Europe.

## Data availability

No data are associated with this article.

## Figures and Tables

**Appendix I.** The SHAPES consortium.

No.	Participant organisation	Short name	Country
1	Maynooth University	MU	IE
2	Access Earth Limited	AELTD	IE
3	AGE Platform Europe	AGE	BE
4	*Associazione Italiana Assisteza Spastici Provincia di Bologna onlus*	AIAS	IT
5	*Aristotelio Panepistimio Thessalonikis –* Aristotle University of Thessaloniki	AUTH	EL
6	*Carus Consilium Sachsen GmbH*	CCS	DE
7	*Clinika de Kay - Clinica Humana*	CH	ES
8	EDGENEERING *Lda*	EDGE	PT
9	European Union of the Deaf aisbl	EUD	BE
10	*Fakultni Nemocnice Olomouc* – University Hospital Olomouc	FNOL	CZ
11	*Fraunhofer Gesellschaft zur Foerderung der Angewandten Forschung E.V.*	FhG	DE
12	Future Intelligence Limited	FINT	CY
13	GNOMON Informatics SA	GNO	EL
14	*Institut für Gesundheitswirtschaft e.V. (gewi)*	GEWI	DE
15	Intracom SA Telecom Solutions	ICOM	EL
16	KOMPAI Robotics	KOM	FR
17	Laurea University of Applied Sciences Ltd	LAUREA	FI
18	MedicalSyn GmbH	MedSyn	DE
19	Northern Health and Social Care Trust	NHSCT	UK
20	Omnitor AB	OMN	SE
21	*Palacký* University Olomouc – Social Health Institute	UP	CZ
22	PAL Robotics SL	PAL	ES
23	Regional Health Authority of Central Greece	DYPE	EL
24	*Residencia de Mayores el Salvador – Asociatión Benéfico-Social el Salvador*	SAL	ES
25	Rock Couture Productions Ltd – Agile Ageing Alliance	AAA	UK
26	Science for You – *Epistimi Gia Sena Astiki Mi Kerdoskopiki Etairia*	SciFY	EL
27	Hellenic Mediterranean University	HMU	EL
28	Tree Technology SA	TREE	ES
29	*Universidad de Castilla - La Mancha*	UCLM	ES
30	*Universidade de Aveiro* – Digital Media and Interaction Research Centre	UAVR	PT
31	*Universidade do Porto*	UPORTO	PT
32	University College Cork	UCC	IE
33	University of Nicosia Research Foundation	UNRF	CY
34	University of Ulster	ULS	UK
35	VICOMTECH	VICOM	ES
36	World Federation of the Deafblind	WFDB	NO

## Footnotes

^1^Technological Readiness Level (TRL) 9: Actual system proven in operational environment (competitive manufacturing in the case of key enabling technologies; or in space).

^2^Other projects in the Health and Care Cluster are: Activage: Internet of Things for ageing well; Adlife: integrated personalised care for advanced chronic patients; Faith: a federated artificial intelligence solution for monitoring mental health status after cancer treatment; GateKeeper: Smart data driven solutions for personalized early risk detection and intervention; InteropEHRate: EHR in people’s hand across Europe; Pharaon: pilots for active and healthy ageing; Smartbear: smart big data platform to offer evidence-based personalised support for healthy and independent living at home.
