# Supplementary material for: Implementation of a pan-European ecosystem and an interoperable platform for Smart and Healthy Ageing in Europe: An Innovation Action research protocol
Source: Open Res Eur. 2022 Jul 4;2:85. [Version 1] doi: 10.12688/openreseurope.14827.1 (PMC10446093; doi:10.12688/openreseurope.14827.1)
Supplement: Peer review history

# Peer review: Reviewer response for version 1

**DOI:** 10.21956/openreseurope.16016.r31470

**Published:** 2023-07-27

**Authors:** Vanessa Zorrilla-Muñoz

**Affiliations:** 1 Bioengineering Institute, Miguel Hernandez University of Elche, Elche, Valencian Community, Spain

Thank you very much for your article. I greatly appreciate your research "Implementation of a pan-European ecosystem and an interoperable platform for Smart and Healthy Ageing in Europe: An Innovation Action research protocol" and am very grateful to be able to review it. Here are some suggestions to improve it and I hope that all this can facilitate a more visible article from an interdisciplinary approach:

1. The partners are described in detail, but the activities in which each of them will work have not been specified.
2. It would help if, in the introduction, when talking about the topics, the objectives that will be worked on in SHAPE were included and, in turn, related to the WP. All of this, in turn, could be connected to the sustainable development goals.
3. The titles "Understanding older adults, their lifeworld, and social contexts" and etc. they should be listed in Figure 3 as objectives (or mention the objectives in another table as I have already commented). In this way, I suggest that the WP be mentioned with the full name so as not to have to constantly look at the table. The WPs could be developed in an orderly fashion, instead of mixing the description, separating paragraphs and ignoring the following WPs, that is, concentrating on the description, sample and detail of who is going to carry out said activities in each WP.
4. In the ethics of activities and, given the possibility of developing a completely transferable system, the possibility of a medical device must also be considered. In this sense, it would be necessary to apply and comment on the ethics of medical devices, as well as all the regulatory requirements and aspects.
5. The architecture of Figure 5. generically comments on market aspects, but feedback through the health product is not reflected. For this reason, it is also convenient to mention not only the layout of the database, but also how it can be suggested through new technological tools related to the topics discussed in the introduction. The contribution and possible technological transfer, as well as the TRL that the system could reach, once the investigation is finished. All this can be connected with the section "Market shaping to inform SHAPES' business models and socio-economic impact" and Figure 6. Instead of commenting on market aspects (since they could correspond to future phases that are not scope in this project) mention should be made of TRLs upon completion. On the other hand, if despite everything you want to continue commenting on market aspects, you should mention real indicators, for example, CARG, demand, etc... instead of replicating the theory at the system, meso, macro level, and micro, which is discussed in the social literature. In short, it is necessary to consider critical market, economic and socioeconomic indicators, as well as technological ones.
6. The risks of the project do not appear at any time. A risk analysis would be useful as a form of evaluation to achieve the intended objectives. A risk analysis is suggested from market aspects (financial, technological, economic, regulatory...), not only social.

Is the study design appropriate for the research question?

Yes

Are sufficient details of the methods provided to allow replication by others?

Partly

Is the rationale for, and objectives of, the study clearly described?

Yes

Are the datasets clearly presented in a useable and accessible format?

Partly

Reviewer Expertise:

Economics and Markets, Technology, Engineering, Sustainability, Sociology.

I confirm that I have read this submission and believe that I have an appropriate level of expertise to confirm that it is of an acceptable scientific standard.

# Peer review: Reviewer response for version 1

**DOI:** 10.21956/openreseurope.16016.r33655

**Published:** 2023-07-18

**Authors:** Qiping Fan

**Affiliations:** 1 Department of Public Health Sciences, Clemson University, Clemson, South Carolina, USA; 2 Department of Health Behavior, School of Public Health, Texas A&M Health Science Center, Texas A&M University, Texas, USA

This protocol article reported the SHAPES project's objectives, methods, structure, and expected outcomes. The article provides a comprehensive SHAPSE Consortium framework and structure of the projects and described stakeholder engagement well. The evaluation plans are well-rounded in user case-level, theoretical aspects, and systematic levels. 

 I only have a few comments for the authors to consider:

1. Under the evaluation methods, please elaborate on the tools/instruments that would be used to evaluate the key indicators.  
2. Please discuss possible influences of internal and external events that may happen in designated field sites.
3. Please discuss plans to maintain the sustainability of the project.  

Is the study design appropriate for the research question?

Yes

Are sufficient details of the methods provided to allow replication by others?

Yes

Is the rationale for, and objectives of, the study clearly described?

Yes

Are the datasets clearly presented in a useable and accessible format?

Yes

Reviewer Expertise:

Public health sciences

I confirm that I have read this submission and believe that I have an appropriate level of expertise to confirm that it is of an acceptable scientific standard.

# Peer review: Reviewer response for version 1

**DOI:** 10.21956/openreseurope.16016.r31471

**Published:** 2023-06-06

**Authors:** Ella Cohn-Schwartz

**Affiliations:** 1 Department of Epidemiology, Biostatistics, and Community Health Sciences, Ben-Gurion University of the Negev, Be'er Sheva, South District, Israel

The paper presents an impressive project spanning a large range of experts from different backgrounds and countries, aiming to create an open pan-European ecosystem including a standardised, interoperable, and scalable platform for the integration of smart technologies. This platform can be used to promote the health and well-being of older adults. The project seems to be making good progress, despite a rocky start due to the COVID-19 pandemic. The project is adequately described in detail, and I only have a few small comments.

1. WP2 aims to explore the lifeworlds of older individuals, and it could be useful to specify whether specific attention will be paid to the use of technology by these adults, since this is the topic of the current project.
2. The difference between WP4 and WP5 could be made clearer.
3. When describing WP5, the authors might want to mention whether older adults themselves will be involved in the UX development process, at which stages and to what extent.
4. The authors might want to add to the "Dissemination of results" section the address of the project website and some examples of published papers from the project.

Is the study design appropriate for the research question?

Yes

Are sufficient details of the methods provided to allow replication by others?

Yes

Is the rationale for, and objectives of, the study clearly described?

Yes

Are the datasets clearly presented in a useable and accessible format?

Yes

Reviewer Expertise:

Aging adults (gerontology), social relationships at old age, cognitive aging, ageism.

I confirm that I have read this submission and believe that I have an appropriate level of expertise to confirm that it is of an acceptable scientific standard.
